# Optimized culture protocol for improved isolation of nontuberculous mycobacteria from skin biopsy^؆^

**DOI:** 10.1016/j.abd.2026.501379

**Published:** 2026-06-15

**Authors:** Lucas Evangelista Marques, Rômulo Henrique Malaquias, Stéphanie Chrystine Balestro Mota, Hélio Amante Miot, Samira Yarak, Cristina Viana-Niero

**Affiliations:** a Department of Microbiology, Immunology and Parasitology, Escola Paulista de Medicina, Universidade Federal de São Paulo, São Paulo, SP, Brazil; b Department of Dermatology, Escola Paulista de Medicina, Universidade Federal de São Paulo, São Paulo, SP, Brazil; c Department of Infectology, Dermatology, Imaging Diagnosis and Radiotherapy, Faculty of Medicine, Universidade Estadual Paulista, Botucatu, SP, Brazil

Dear Editor,

Nontuberculous Mycobacteria (NTM) are ubiquitous in the environment and comprise more than 190 species, many of which demonstrate intrinsic resistance to conventional antimicrobials.^1^ In dermatologic practice, these organisms have become important etiologic agents of cutaneous and subcutaneous infections, particularly following aesthetic and surgical procedures. The most frequently implicated species ‒ *Mycobacterium abscessus*, *M. chelonae*, and *M. fortuitum* ‒ are rapidly growing mycobacteria associated with infection cases and outbreaks.^2^ Clinically, NTM skin infections are often indolent and polymorphic, presenting as erythematous nodules, abscesses, or chronic ulcers that mimic other infectious or inflammatory dermatoses.^3^, ^4^ Due to this nonspecific presentation, patients are frequently treated empirically with antibiotics directed against pyogenic bacteria, which can delay the correct diagnosis. Repeated therapeutic failures permit the infection to advance, potentially leading to chronicity and deeper tissue involvement.^5^

Definitive diagnosis relies on microbiological confirmation, ideally through mycobacterial culture from skin biopsy specimens or exudates, which enables species identification and antimicrobial susceptibility testing.^6^ However, culturing NTM from skin tissue remains challenging due to low bacillary load, prior antibiotic exposure, and the absence of standardized protocols for sample decontamination for cultivation. Moreover, granulomatous inflammation with scarce exudation ‒ typical of cutaneous mycobacterioses ‒ further limits bacterial recovery. Consequently, optimizing decontamination and culture methods adapted to dermatologic specimens is crucial to improve diagnostic yield and guide therapy in NTM skin infections.

In this context, the present study evaluated and compared decontamination protocols to enhance the isolation of NTM from skin biopsy specimens. We propose a reproducible and feasible methodology suitable for routine diagnostic laboratories, aiming to shorten diagnostic delays and improve clinical management of cutaneous NTM infections.

This study was conducted between December 2023 and September 2025 at the Dermatology Clinic of the Hospital São Paulo ‒ UNIFESP (HSP-UNIFESP), Brazil. The study included adult patients (≥ 18-years) with clinical suspicion of cutaneous infection by NTM following aesthetic procedures with injected biomaterials. Eligible participants presented with persistent erythematous or violaceous papules, nodules, plaques, or ulcers, and had shown poor or no response to prior empirical antibiotic monotherapy. A total of 11 skin biopsy specimens were obtained from 11 patients under sterile conditions using a 5 mm punch. Each specimen was divided for parallel microbiological and histopathological analysis. For microbiological processing, fragments were transferred to sterile vials, maintained at 4 °C, and processed within four hours. All biopsy specimens were processed in three laboratories specialized in mycobacteriology: Laboratory of Mycobacterial Molecular Biology (LMMB), Central Laboratory of HSP (LC), and External Laboratory (LF). At the LMMB, the experimental protocol was applied to all samples using 0.1% Cetylpyridinium Chloride (CPC-Synth), Fig. 1. The final sediment was inoculated onto Middlebrook 7H9 and 7H10 media supplemented with oleic acid-albumin-dextrose-catalase and PANTA (40 U/mL polymyxin-B, 4 µg/mL amphotericin-B, 16 µg/mL nalidixic acid, 4 µg/mL trimethoprim, and 4 µg/mL azlocillin) (Becton Dickinson) and incubated at 30 °C for up to 60-days. In the remaining laboratories, specimens were decontaminated using the Petroff (4% NaOH) and N-Acetyl-L-Cysteine (NALC-NaOH) methods, respectively, and subsequently inoculated onto Löwenstein-Jensen medium or into Mycobacteria Growth Indicator Tubes, according to national guidelines.^7^

**Figure 1:**
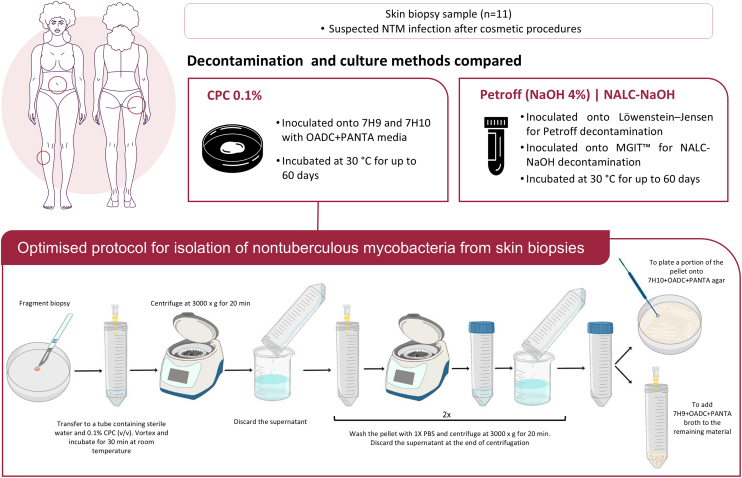
Comparative analysis of decontamination and culture methods for the isolation of Nontuberculous Mycobacteria (NTM) from skin biopsies. The upper panel illustrates the conventional methods used for comparison: Petroff (NaOH 4% on Löwenstein-Jensen) and NALC-NaOH (on MGIT). The lower panel details the proposed optimized protocol using CPC 0.1%, including the fragmentation of the biopsy, incubation for 30 minutes, sequential washing with PBS, before final inoculation into 7H10 agar and 7H9 broth supplemented with OADC and PANTA.

Colonies obtained from each culture were evaluated by Ziehl-Neelsen staining and phenotypic characteristics. Species identification was performed by Matrix-Assisted Laser Desorption/Ionisation Time-Of-Flight (MALDI-TOF) mass spectrometry using a Microflex LT-Bruker Daltonics and scores ≥ 1.8.^8^

Analyses were concurrently performed by independent mycobacteriology laboratories for culture and species identification (Table 1). The CPC-based protocol successfully isolated mycobacteria from 5 of 11 skin biopsies (45%). In comparison, the LC laboratory recovered mycobacteria from only 1 of 8 samples (12.5%), and no isolates were obtained at LF. Histopathological examination revealed granulomatous inflammation in all samples, with or without foreign-body reaction. Acid-fast bacilli were identified in two samples (18%). Based on clinical, histopathological, and microbiological correlation, cases were classified as mycobacterial infection (n = 5) or non-infectious inflammatory processes (n = 6).

**Table 1:** Summary of biopsy types, biomaterial, and corresponding microbiological and histopathological findings in clinical samples.

ID	Biopsy site	Material	LBMM	LC Petroff/LJ	LF NALC/MGIT/LJ	Maldi-TOF MS (score)	Histopathological features
1	Abdomen	Injection lipolysis (non-specified agents)	+	‒	NR	*M. fortuitum* (2.256)	Chronic granulomatous inflammation with necrosis; AFB positive.
2	Jaw	Polymethyl methacrylate and silicone oil	+	NR	‒	*M. chelonae* (2.064)	Foreign body granuloma (PMMA/silicone); AFB negative.
3	Buttocks	Hyaluronic acid	+*	‒	NR	*M. fortuitum* (2.020)	Nonspecific ulcerated lesion with fibrosis; AFB negative.
4	Face	Calcium hydroxyapatite	+	‒	NR	*M. fortuitum* (2.093)	Suppurative granulomatous dermatitis; AFB negative.
5	Face	Poly-l-lactic acid	+	+	NR	*M. abscessus* (2.216)	Tuberculoid-type granulomatous dermatitis; AFB positive.
6	Face	Hyaluronic acid	‒	‒	NR	‒	Tuberculoid-type granulomatous dermatitis; AFB negative.
7	Jaw	Hyaluronic acid	‒	‒	NR	‒	Chronic granulomatous inflammatory process with superficial and deep foreign-body-type gyant cell reaction.
8	Nose	Nasal thread lift using nylon sutures	‒	‒	NR	‒	Fibrosis; NOTE: Serial sectioning at three deeper levels was performed and the histologic appearance remained unchanged.
9	Leg	Silicone oil	‒	‒	NR	‒	Foreign body granuloma with fibrosis and dystrophic calcification; AFB negative.
10	Buttocks	Injection lipolysis (non-specified agents)	‒	NR	‒	‒	Foreign body granuloma with dermal fibrosis; AFB negative.
11	Eyelid	Silicone	‒	NR	‒	‒	Foreign body granulomatous inflammation with lobular panniculitis; multivacuolated macrophages; no tuberculoid granulomas; AFB negative.

+, Growth in 7H10 e 7H9 medium enriched with OADC-PANTA; +*, Growth only in 7H9 medium enriched with OADC-PANTA; NR, Not performed.

Among confirmed mycobacterial infections, the recovery rate was 100% using the CPC protocol, compared with 25% (1/4) with the Petroff method, and NALC-NaOH did not recover any NTM. All isolates were acid-fast, non-pigmented, and exhibited rapid growth. MALDI-TOF mass spectrometry identified *Mycobacterium fortuitum*, *M. chelonae* and *M. abscessus* with high-confidence scores (> 2.0), corroborating phenotypic characteristics, Table 1 and Fig. 2.

**Figure 2:**
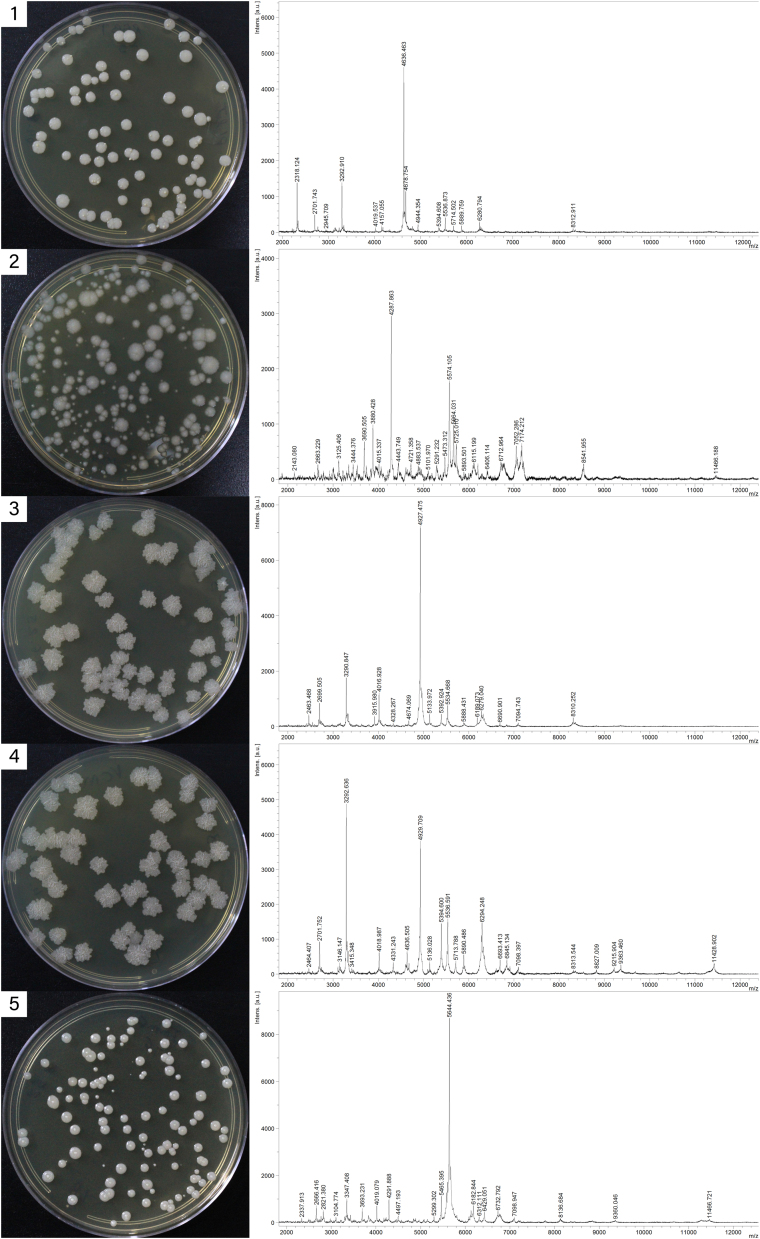
Phenotypic and molecular characteristics of nontuberculous mycobacteria (NTM) clinical isolates. The left panel (1–5) displays the macromorphological characteristics of colonies on 7H10 agar, while the right panel presents the corresponding MALDI-TOF MS spectra of the isolates, identified as *M. fortuitum* (1, 3, and 4), *M. chelonae* (2), and *M. abscessus* (5). All species were successfully recovered from skin biopsy samples using the proposed optimized 0.1% CPC protocol.

Our findings revealed clear differences in recovery according to the protocol employed. The CPC-based method achieved successful isolation in all confirmed cases (5/5), whereas Petroff recovered one of four, and NALC-NaOH none (0/1). Although limited by sample size, this pattern highlights the influence of chemical choice on culture performance. The superior yield with CPC likely reflects its mild bacteriostatic detergent effect, which increases cell-wall permeability and preserves the viability of mycobacteria protected by mycolic acids.^9^ In contrast, NaOH ‒ used in both Petroff and NALC protocols ‒ is a strong alkali capable of diffusing through bacterial porins, disrupting intracellular pH, and inducing DNA and membrane damage, thereby exerting a bactericidal effect proportional to concentration and contact time.^10^

Comparable studies of cutaneous NTM infections that applied grinding processing by burnisher treatment or decontamination using NALC-NaOH presented a culture positivity rate ranging from 66% to 80%.^11^ In a recent Peruvian case series of NTM infections following aesthetic procedures, culture-confirmed NTM were recovered in 3/7 of cases (42%), despite all samples being Ziehl-Neelsen positive, underscoring persistent diagnostic challenges.^12^ These findings collectively reinforce our observation that the choice of decontamination method significantly influences culture outcomes in skin specimens, and support a shift toward milder, tissue-appropriate protocols.

Another determinant of successful recovery of NTM was the use of antibiotic-supplemented culture medium. LBMM, which combined mild decontamination with cultivation in Middlebrook 7H9 and 7H10 media supplemented with PANTA, achieved recovery in all positive cases, including one detected exclusively in 7H9 + PANTA. In contrast, laboratories applying NALC or Petroff decontamination followed by cultivation in MGIT or Löwenstein-Jensen media obtained limited or null recovery. Partial concordance between laboratories in three samples suggests that both decontamination and culture conditions jointly determine diagnostic yield.

The small number of biopsies (11 specimens from 11 patients) represents a limitation inherent to the rarity of cutaneous NTM infections and to logistical constraints in single-center studies. Furthermore, incomplete pairing of samples across laboratories precluded robust statistical testing. Despite these limitations, the present data suggest that CPC-based decontamination provides higher recovery from skin biopsies than conventional NaOH-based methods. Implementing such a protocol could reduce diagnostic delays, enable earlier targeted therapy, and prevent chronic sequelae such as deep-tissue extension and scarring ‒ particularly relevant in procedure-related infections.

Future investigations should validate the CPC protocol in larger, multicenter, fully paired cohorts. Evaluating the impact of laboratory-specific variables such as staff expertise, media formulation, and incubation temperature may further refine standard operating procedures. Ultimately, standardization of NTM culture methodologies for cutaneous specimens is essential to improve diagnostic accuracy and therapeutic outcomes in patients with mycobacterial skin disease.

In conclusion, CPC-based protocol improved the recovery of NTM from skin biopsy specimens. This simple, reproducible method is feasible for implementation, supporting faster species identification and improved therapeutic management.

## ORCID ID

Lucas Evangelista Marques: 0000-0003-2597-5149

Rômulo Henrique Malaquias: 0009-0005-2646-2785

Stéphanie Chrystine Balestro Mota: 0000-0001-7540-9650

Hélio Amante Miot: 0000-0002-2596-9294

Cristina Viana-Niero: 0000-0002-1068-7884

## Ethical approval

The study protocol was approved by the Research Ethics Committee of HSP-UNIFESP, under protocol number CAAE 91309425.2.1001.5505.

## Data availability statement

The data that support the findings of this study are available from the corresponding author upon reasonable request.

## Research data availability

Does not apply.

## Financial support

The first author of this paper receives funding from the Brazilian Agency for the Support and Evaluation of Graduate Education (CAPES) – Funding Code 001.

## Authors' contributions

Lucas Evangelista Marques: Contributed to the study concept and design; performed laboratory investigations; organized and analyzed the data, and wrote the original draft of the manuscript, in addition to approving the final version.

Rômulo Henrique Malaquias: Contributed to clinical data collection, analysis and interpretation; participated in patient recruitment and clinical evaluation, and critically revised the manuscript; approving its final version.

Stéphanie Chrystine Balestro Mota: Participated in laboratory processing, data curation, and manuscript revision, and approved the final version.

Hélio Amante Miot: Contributed to statistical analysis; critical review of the literature and manuscript review, and approved the final version.

Samira Yarak: Supervised and participated in patient recruitment and clinical evaluation; contributed to clinical data analysis and manuscript review, and approved the final version.

Cristina Viana-Niero: Designed the methodology; supervised microbiological procedures and data analysis; reviewed the manuscript, and approved the final version.

## Conflicts of interest

None declared.
